# A Self-Sensing Active Magnetic Bearing Based on a Direct Current Measurement Approach

**DOI:** 10.3390/s130912149

**Published:** 2013-09-11

**Authors:** Andries C. Niemann, George van Schoor, Carel P. du Rand

**Affiliations:** School of Electrical, Electronic and Computer Engineering, North-West University, 11 Hoffman Street, Potchefstroom 2531, South Africa; E-Mails: aniemann@csir.co.za (A.C.N.); george.vanschoor@nwu.ac.za (G.S.)

**Keywords:** self-sensing, active magnetic bearing (AMB), direct current measurement (DCM), position estimation, magnetic suspension, duty cycle

## Abstract

Active magnetic bearings (AMBs) have become a key technology in various industrial applications. Self-sensing AMBs provide an integrated sensorless solution for position estimation, consolidating the sensing and actuating functions into a single electromagnetic transducer. The approach aims to reduce possible hardware failure points, production costs, and system complexity. Despite these advantages, self-sensing methods must address various technical challenges to maximize the performance thereof. This paper presents the direct current measurement (DCM) approach for self-sensing AMBs, denoting the direct measurement of the current ripple component. In AMB systems, switching power amplifiers (PAs) modulate the rotor position information onto the current waveform. Demodulation self-sensing techniques then use bandpass and lowpass filters to estimate the rotor position from the voltage and current signals. However, the additional phase-shift introduced by these filters results in lower stability margins. The DCM approach utilizes a novel PA switching method that directly measures the current ripple to obtain duty-cycle invariant position estimates. Demodulation filters are largely excluded to minimize additional phase-shift in the position estimates. Basic functionality and performance of the proposed self-sensing approach are demonstrated via a transient simulation model as well as a high current (10 A) experimental system. A digital implementation of amplitude modulation self-sensing serves as a comparative estimator.

## 1. Introduction

Active Magnetic Bearings (AMBs) permit frictionless suspension of the rotor through magnetic forces, rendering them a key technology for various industrial applications [1]. AMBs most frequently use dedicated non-contact displacement sensors to provide position feedback. In the continued drive to reduce hardware complexity and production costs, manufacturers of AMBs aim to produce compact integrated systems that are more reliable and economical.

Self-sensing facilitates rotor position estimation by consolidating the sensing and actuating functions into a single electromagnetic transducer. In magnetic bearings, the stator coil electrical inductance is influenced by the displacement of the rotor within the air gap [1,2]. Position feedback control is therefore accomplished using the measured coil currents and voltages to estimate rotor displacement.

The general agreement in the literature is that self-sensing research can be grouped into two main categories [1–4]. The first category considers a linear time invariant (LTI) process model in the estimation algorithm. In this methodology, a classical LTI state-observer generates estimates of the rotor position from the coil voltage and current measurements [1]. In the work by [5] a linear state-space observer was used to estimate the rotor position. It was shown that the state model is observable from the current measurement only. The drawbacks of the methods in this category are low robustness, difficulty to realize feedback stabilization, and high sensitivity to parameter variations [1,3–6]. The second category includes nonlinear or linear time varying process models. Applying a periodic perturbation to the plant will result in a linear time periodic (LTP) system exhibiting improved self-sensing performance [7,8]. Since most industrial AMBs use high efficiency switching power amplifiers (PAs), periodic perturbations (*i.e.*, switching ripples) are inherently present in the coil currents. The ripple component can then be employed in modulation techniques to estimate rotor position. The main advantages of this approach are improved system robustness, uncoupled sensing information at high frequencies, and minimal additional hardware requirements. In the literature, a large part of research focuses on this solution for self-sensing AMBs [1–4,7–11].

Amplitude demodulation techniques inherently involve the use of band-pass (BPF) and low-pass (LPF) filters to isolate and manipulate the high frequency fundamental components (voltage and current) for position estimation [3]. However, these filters introduce additional phase-shifts that result in lower stability margins. Furthermore, the demodulated position estimate is duty-cycle dependent since the current ripple amplitude is nonlinearly modulated when the duty-cycle changes [2,3]. To compensate for the duty-cycle variation, demodulation techniques use a nonlinear observer considering the bearing coil model [12], or the demodulated voltage divides the demodulated current [3].

This paper extends the work presented in [3] and addresses the aforementioned problems via the direct current measurement (DCM) approach for self-sensing AMBs, where DCM refers to the direct measurement of the ripple current component. What sets the work of [3] and the method proposed in this paper apart from other work in the self-sensing literature is the inclusion of magnetic nonlinearity. The proposed self-sensing mechanism employs a novel PA switching method that only measures the peak current ripple to obtain duty-cycle invariant position estimates (single-input single-output system). Since the DCM approach largely excludes demodulation filters, additional phase-shift in the position estimates are minimized. The results are compared with a digital replica of the modulation self-sensing algorithm proposed in [3] (the system in [3] comprises analog demodulation filters and a 1.2 A decoupled AMB). The practical implementation of a high current (10 A) AMB employing DCM self-sensing and classical position and current control demonstrates its feasibility for industrial application.

The contents of this paper are organized as follows: Section 2 presents the underlying modeling principles of the DCM self-sensing approach. The reference transient simulation model, the high current experimental heteropolar AMB, and the practical implementations of the DCM and digital demodulation algorithms are described in Section 3. Section 4 reports the static and dynamic performance of the self-sensing sensors. Finally, Section 5 summarizes the concluding remarks.

## 2. DCM Self-Sensing

### 2.1. Governing Equations

**Figure 1.**
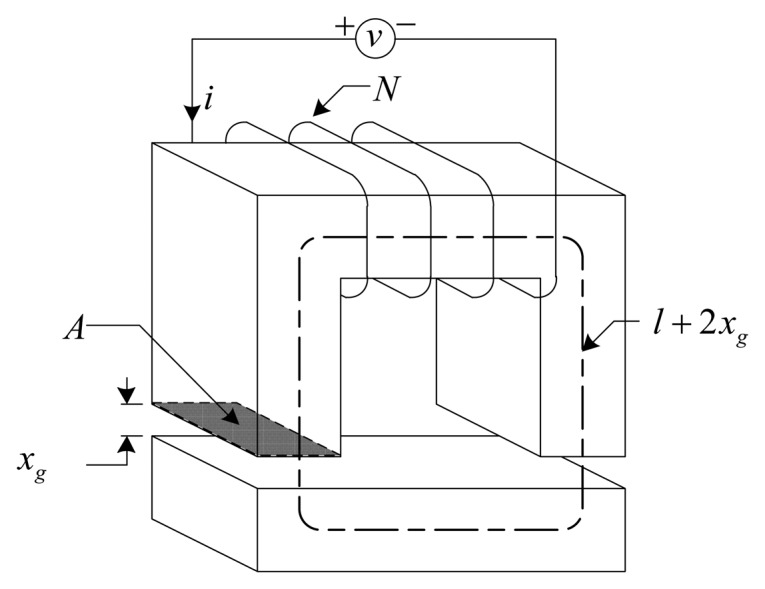
Simplified electromagnetic actuator.

The DCM method exploits the fact that rotor displacement is directly related to the current ripple amplitude during a switching cycle [1,2]. Consider the simplified one degree of freedom (DOF) electromagnetic actuator presented in Figure 1 [4]. The relationship between the voltage (*v*), current (*i*), and position is described by [4]:

(1) $$v(t)=\mu_{0}N^{2}A\left[\frac{1}{2x_{g}(t)+l/\mu_{r}}\frac{di(t)}{dt}-2\frac{i(t)}{{\left(2x_{g}(t)+l/\mu_{r}\right)}^{2}}\frac{dx_{g}(t)}{dt}\right]+i(t)R$$

with *μ*_0_ the permeability of free space, *N* the number of coil turns, *A* the pole face area, *x_g_* the air gap length, *l* the effective magnetic material path length, *μ_r_* the magnetic material relative permeability, and *R* the coil resistance. By neglecting nonlinear magnetic effects as well as coil resistance, and assuming that the movement of the AMB rotor is slow compared to the high frequency coil current, the air gap is described by Equation (2):

(2) $$x_{g}(t)=\left[\frac{\mu_{0}N^{2}A}{2v(t)}\right]\frac{di(t)}{dt}-\frac{l}{\mu_{r}}$$

The DCM self-sensing approach is based on this simplified inductor model, a novel PA switching method, and the least-square algorithm proposed by [3] to address the problem of magnetic material nonlinearity. In general, the voltage ripple is also measured in modulation techniques to compensate for the nonlinear effect of duty-cycle change [2–4].

**Figure 2.**
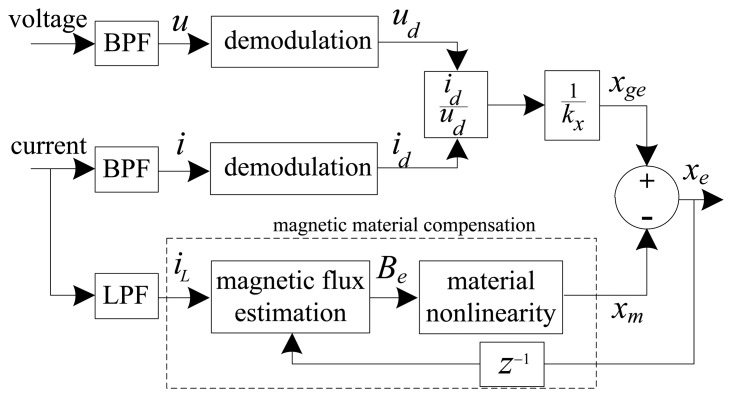
Modulation self-sensing algorithm.

**Figure 3.**
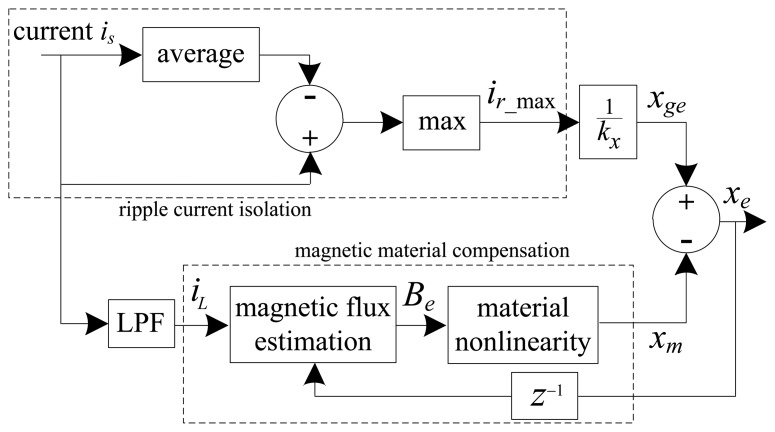
DCM self-sensing approach.

Alternatively, this work proposes a more simplistic approach by measuring the maximum amplitude of the current ripple directly during a constant 50% duty switching cycle (discussed in next section). Due to the constant 50% duty cycle each time the current ripple amplitude is measured, the voltage in Equation (2) becomes constant. Since the switching time is now also fixed, the derivative of the current becomes proportional to the amplitude of the current during the 50% duty cycle. With 1/*μ_r_* modeled by a second order estimation function [3], the position estimate follows from Equation (2) as:

(3) $$x_{e}=x_{ge}-x_{m}=\frac{i_{r\_\max}}{k_{x}}-\frac{1}{k_{x}}\left[k_{2}B_{e}^{2}+k_{1}B_{e}+k_{0}\right]$$

where *x_ge_* is the uncompensated estimated position, *x_m_* the magnetic material compensation term, *i_r__*_max_ the maximum current ripple amplitude, *k_x_* the current to position conversion (scaling) constant, *B_e_* the estimated magnetic flux density in the pole, and *k*_0,1,2_ empirically determined coefficients for the bearing inductor model. In Equation (3)*_e_* and *k*_0,1,2_ realize a 2nd order estimation function to compensate for magnetic material nonlinearities. The parameters of the inductor model *k_x_*, *k*_0,1,2_ are determined via simple experiments as described by [3]. The estimate of the magnetic flux *B_e_* is given by:

(4) $$B_{e}=\left[\frac{\mu_{0}N}{2(x_{e-1}+g_{0})}+\frac{L_{s}}{NA}\right]i_{L}$$

with *x_e-_*_1_ the delayed estimated position (one sample), *g*_0_ the nominal air gap length, *L_s_* the leakage inductance, and *i_L_* the low frequency control current component. Figures 2 and 3 present functional diagrams of the modulation [3] and DCM approaches to highlight their fundamental differences. Figure 3 shows that DCM self-sensing utilizes only the measured raw coil current (*i_s_*). The algorithm does not require demodulation of the current and voltage signals, thereby reducing the number of filters in the position estimation model. The additional phase-shift introduced by these filters ultimately results in lower robustness for AMB control [2,13].

### 2.2. PA Switching and Current Ripple Isolation

**Figure 4.**
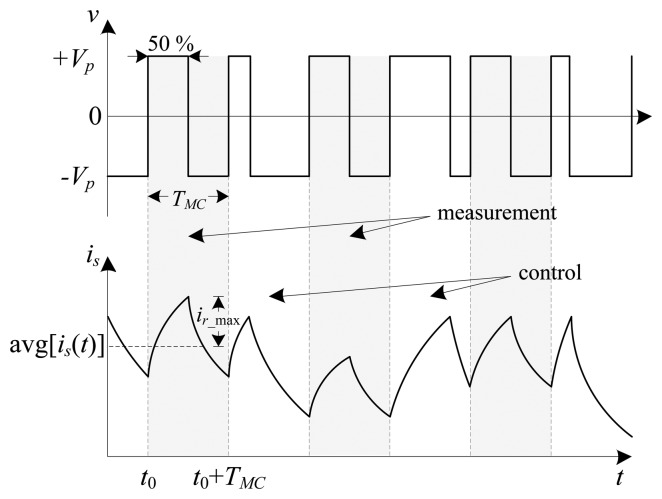
Raw coil voltage and current showing measurement and control cycles.

In [2] it is established that the amplitude of the current ripple is a function of both the bearing coil inductance and the voltage duty cycle. To remove the nonlinear dependency of the estimated position on the duty cycle, the PA switching cycle is constrained to be the same each time the current ripple is measured. Controllability of the system, which requires a varying duty cycle, is met through compromise. The PA switching cycle is divided into alternating measurement (constant) and control (varying) cycles. Figure 4 presents a graphical example of the proposed DCM switching method. The optimal duty cycle for measurement is 50% since the amplitude of the resulting triangular current waveform is an indication of rotor displacement [2]. Furthermore, the nonlinear modulation constant is one for a 50% duty cycle which simplifies the self-sensing algorithm.

Alternating switching cycles are therefore fixed, thus reducing the magnetic bearing's maximum force slew rate. Modulation self-sensing, regardless of the signal-processing algorithm, benefit from limiting the voltage duty cycle to ensure sufficient excitation (*i.e.*, current ripple amplitude), thereby increasing system robustness [2,11,13]. The dynamic performance of an AMB utilizing DCM self-sensing is therefore comparable to that of AMBs employing other self-sensing approaches.

In modulation self-sensing, the high frequency current ripple is isolated by passing the measured current through an analog BPF or high-pass filter (HPF) [2,3,8,9]. This work proposes an alternative technique where the peak ripple current is deduced during a single measurement cycle (*T_MC_*) from the raw coil current waveform by subtracting the average coil current over the whole switching period from the measured raw coil current *i_s_*. The maximum amplitude of the current ripple is given by:

(5) $$i_{r\_\max}=\underset{T_{MC}}{\max}[i_{r}(t)]=\underset{T_{MC}}{\max}\left[i_{s}(t)-\text{avg}(i_{s}(t))\right]$$

with *i_r_* the current ripple component, and avg(*i_s_*(*t*)) the average coil current during the measurement cycle (*i.e.*, the current dc component). However, practical implementation of Equation (5) presents unique challenges in terms of signal resolution. The detailed implementation of the current ripple extraction method is discussed in Section 3.

### 2.3. Algorithm Stability

In order to facilitate a stability analysis of the self-sensing algorithm, the position estimation loop must be linearized. The nonlinear compensation function *f_m_*(*B*) = *k*_2_*B*^2^ + *k*_1_*B* + *k*_0_ is linearized around the nominal low-pass filtered current (*i_L_*_0_) and the nominal rotor position (*g*_0_), given by:

(6) $$f_{m}(i_{L0,g_{0}})=k_{mi}i_{LC}+k_{mx}x$$

with *i_LC_* the current variation around *i_L_*_0_ (*i.e.*, *i_L_* = *i_L_*_0_ + *i_LC_*) and *x* the position variation around *g*_0_. *k_mi_* and *k_mx_* are obtained by the linearization process as:

(7) $$\begin{array}{l} k_{mi}=2k_{2}{\left[\frac{\mu_{0}N}{2g_{0}}+\frac{L_{s}}{NA}\right]}^{2}i_{L0}+k_{1}\left[\frac{\mu_{0}N}{2g_{0}}+\frac{L_{s}}{NA}\right]\\ k_{mx}=2k_{2}\left[\frac{\mu_{0}N}{2g_{0}}+\frac{L_{s}}{NA}\right]\frac{\mu_{0}N}{2g_{0}^{2}}i_{L0}^{2}+k_{1}\frac{\mu_{0}N}{2g_{0}^{2}}i_{L0} \end{array}$$

The linearized position compensation can then be written as Equation (8).

(8) $$x_{m}=\frac{1}{k_{x}}f_{m}(i_{L0,g_{0}})=\frac{k_{mi}}{k_{x}}i_{LC}+\frac{k_{mx}}{k_{x}}x$$

**Figure 5.**
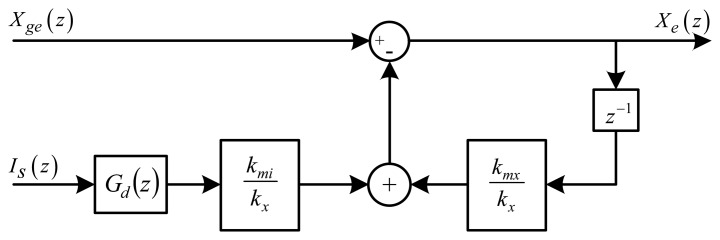
Linearized block diagram (*z*-domain) of self-sensing algorithm.

Since Equation (4) uses a delayed sample of the estimated position, the *z*-transform of Equation (8) is determined. Figure 5 presents a linearized block diagram of the self-sensing algorithm in the *z*-domain, with *G_d_*(*z*) denoting the LPF. The linearized nonlinear compensated position Equation (8) in the *z*-domain is given by Equation (9):

(9) $$X_{m}(z)=\frac{k_{mi}}{k_{x}}I_{s}(z)G_{d}(z)+z^{-1}\frac{k_{mx}}{k_{x}}X_{e}(z)$$

Rewriting Equation (3) using Equation (9), the linearized estimated position is described by Equation (10).

(10) $$\begin{array}{ll} X_{e}(z) & =X_{ge}(z)-X_{m}(z)\\ & =X_{ge}(z)-\frac{k_{mi}}{k_{x}}I_{s}(z)G_{d}(z)-z^{-1}\frac{k_{mx}}{k_{x}}X_{e}(z) \end{array}$$

Since the self-sensing algorithm is dependent on *X_ge_*(*z*) and *I_s_*(*z*), the closed loop transfer functions are given by:

(11) $$T_{X_{ge}}(z)=\frac{X_{e}(z)}{X_{ge}(z)}=\frac{z}{z+k_{mx}/k_{x}}$$

(12) $$T_{I_{S}}(z)=\frac{X_{e}(z)}{I_{s}(z)}=-\frac{K_{mi}}{k_{x}}G_{d}(z)\frac{z}{z+k_{mx}/k_{x}}$$

with *I_s_*(*z*) = 0 in Equation (11) and *X_ge_*(*z*) = 0 in Equation (12).

In Equation (11), the characteristic equation is *λ* = *z* + *k_mx_*/*k_x_*. The self-sensing algorithm will always be stable if the pole given by the ratio *k_mx_*/*k_x_* is inside the unity circle. Furthermore, the input *X_ge_*(*z*) is bounded due to the restricted duty cycle, resulting in a stable algorithm. The characteristic equation in Equation (11) also describes Equation (12). The analysis is similar to Equation (11), given that *G_d_*(*z*) is bounded. Since the LPF is designed to be stable, and *I_s_*(*z*) is restricted due to the limits of the PA, the algorithm is stable. The characteristic equation analyses of Equations (11) and (12) show that *k_mx_*/*k_x_* must be less than one to facilitate Schur stability [14].

### 2.4. Duty Cycle Change Magnetic Cross-Coupling

The poles of a heteropolar AMB are coupled magnetically through the rotor and stator back iron, as well as leakage flux [15]. Magnetic cross-coupling can be reduced by physically separating the individual actuators in the AMB stator. This, however, drastically increases manufacturing costs. Note that although the self-sensing technique is applied in one DOF, the experimental AMB is fully suspended during dynamic evaluation. The results therefore include the effects of magnetic cross-coupling between the AMB poles.

This section presents the effects of magnetic cross-coupling due to a 50% measurement cycle. Using Faraday's law, the current in coil 1 (top actuator) is determined by:

(13) $$i_{1}(t)=\frac{v_{1}(t)}{R}-\frac{N}{R}\frac{d\phi_{1}(t)}{dt}$$

with *R*, *N*, and *ϕ*_1_ the coil resistance, number of coil turns, and the magnetic flux in coil 1 respectively. Rewriting Equation (13) in terms of magnetic fluxes and mutual inductances, the current in coil 1 is given by Equation (14).

(14) $$i_{1}(t)=\frac{v_{1}(t)}{R}-\frac{N}{R}\left[\frac{d\phi_{1}(t)}{dt}+\frac{d\phi_{2}M_{\left(1,2\right)}(t)}{dt}+\frac{d\phi_{3}M_{\left(1,3\right)}(t)}{dt}+\frac{d\phi_{4}M_{\left(1,4\right)}(t)}{dt}\right]$$

with *M*_(1,_*_n_*_)_ the mutual inductance between coil 1 and coils *n* = 2,3,4.

In Equation (14), duty cycle variations will change the sum of the magnetic fluxes, which influences the current ripple gradient. Furthermore, different AMB coil duty cycles will couple onto the sensing current ripple, which is dependent on the mutual coupling constant. By switching all the coils at a constant duty cycle, the effect of magnetic cross-coupling on the position estimate is kept constant. The air gap variation, however, still influences the mutual inductance constants. An example of magnetic cross-coupling due to a 50% measurement cycle is presented in Section 4.

## 3. Reference Models

### 3.1. Transient Simulation Model

**Figure 6.**
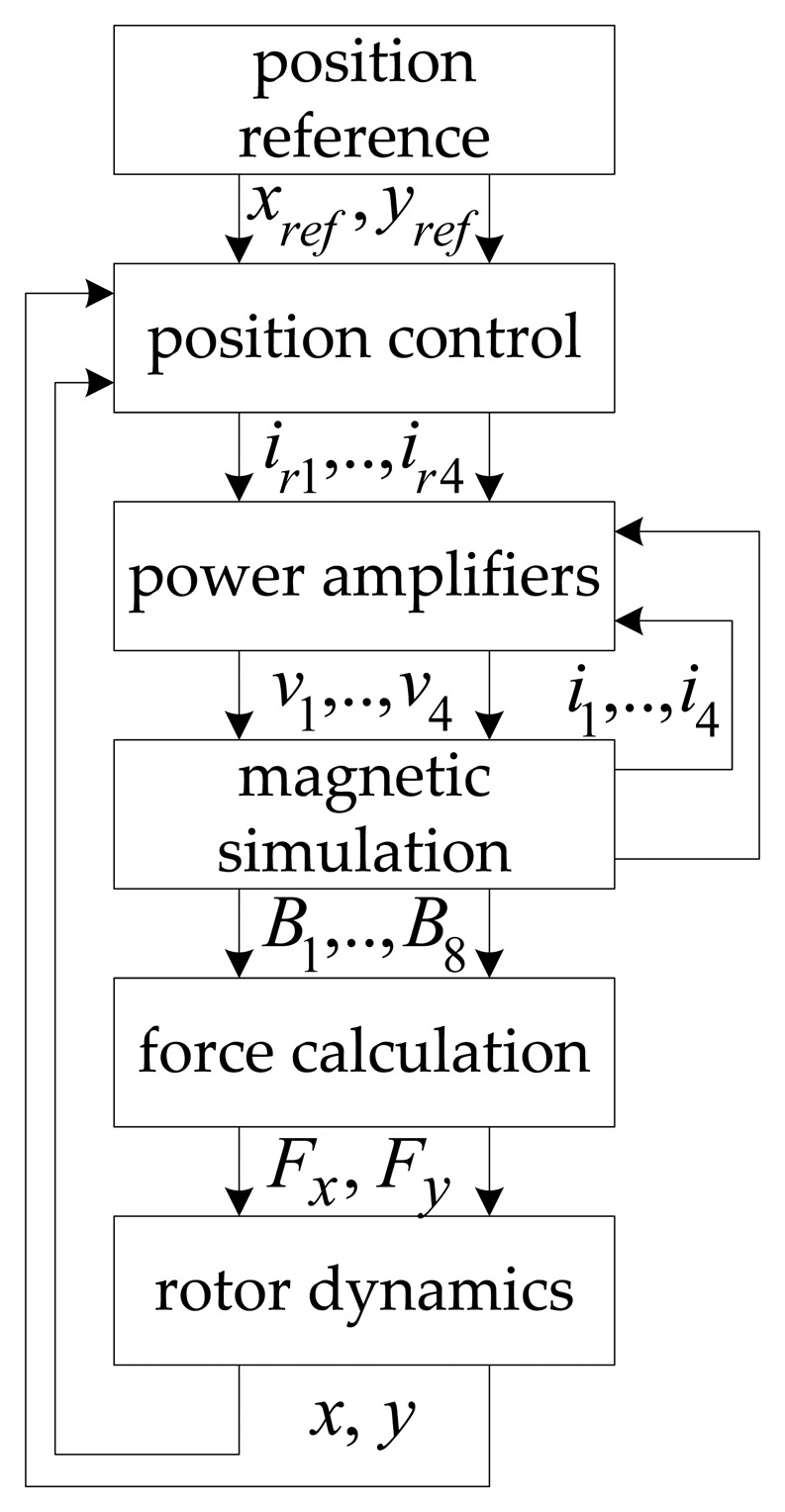
Flow diagram of the transient simulation model.

The accuracy of the self-sensing simulations is dependent on the comprehensiveness of the AMB model. An experimentally verified transient simulation model (TSM), which includes nonlinear effects such as magnetic hysteresis, material saturation, eddy currents, and cross-coupling, is adopted to emulate the experimental system. The flow diagram of the TSM implemented in simulation is shown in Figure 6. Details regarding the individual TSM modules are presented in [16,17].

In Figure 6, the TSM receives *x*-and *y*-axis position references, which are compared to the actual rotor position. The position controllers generate current references for the voltage mode current controlled PAs. Next, the magnetic model receives the PA voltage signals to determine the bearing coil currents as well as the magnetic fluxes. Current signal feedback facilitates PA current control. The resulting *x* and *y* rotor forces are determined using the magnetic fluxes. Finally, the movement of the rotor is modeled by applying the magnetic fluxes to a point mass model.

### 3.2. Experimental AMB System

**Figure 7.**
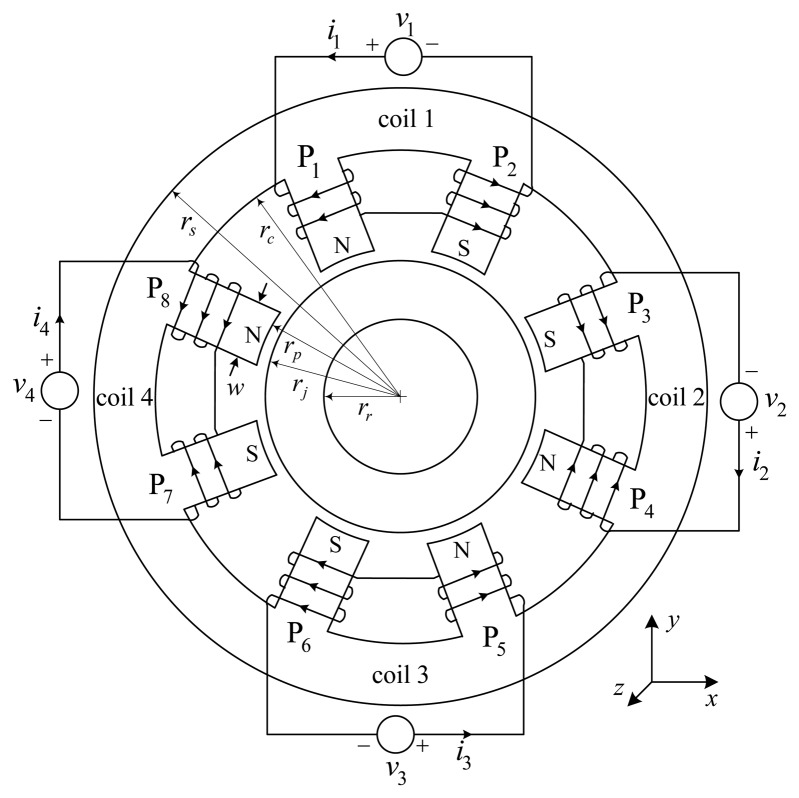
Geometry of an 8-pole heteropolar magnetic bearing.

**Figure 8.**
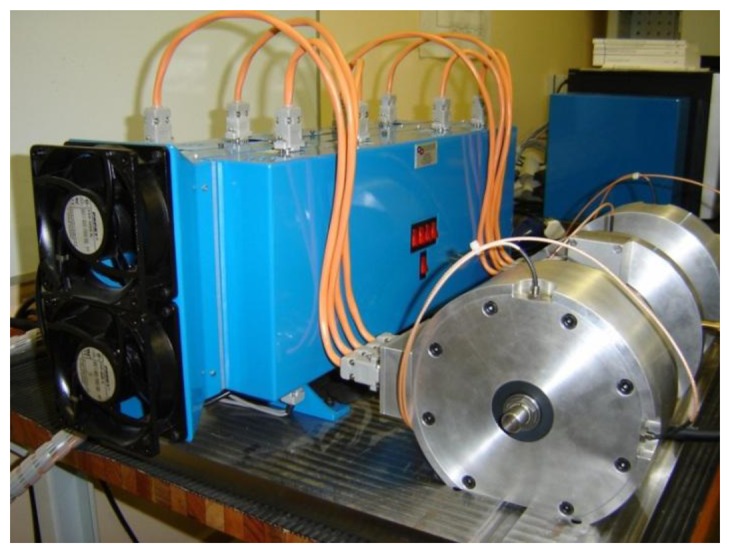
Experimental double heteropolar AMB.

The DCM self-sensing approach is evaluated via an 8-pole heteropolar AMB with referencing geometry shown in Figure 7. A bearing coil denotes adjacent poles that are paired by connecting their respective coils in complementing polarity. Figure 8 shows the experimental radial double heteropolar AMB. The system comprises heteropolar magnetically coupled bearings, a 7.7 kg, 0.5 m flexible rotor, reference eddy-current displacement sensors, and independent high current-controlled PAs. The PAs are configured in two state switch-mode (+*V_p_*, −*V_p_*) in order to ensure high frequency ripple which increases the robustness of the self-sensing AMB [1]. Important bearing and self-sensing parameters are summarized in Table 1.

**Figure 9.**
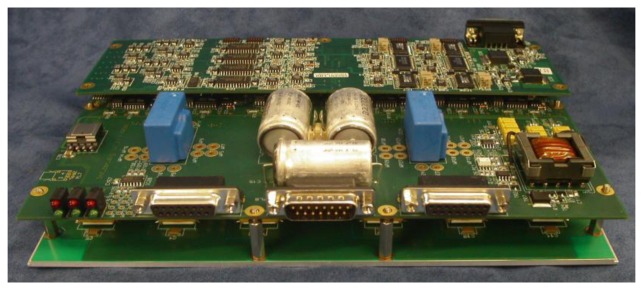
Integrated self-sensing power amplifier module.

A compact integrated PA is designed in-house. The system accommodates the self-sensing scheme, position and current controllers, as well as the measurement and PA electronics. The power electronics implement two full H-bridge configurations, thereby realizing suspension of the AMB rotor in one DOF via a single PA module. The integrated system is shown in Figure 9. Although a bespoke design is used for the amplifiers, commercial switch-mode PAs can be applied by adjusting the PWM control routine in software.

### 3.3. DCM Self-Sensing Implementation

**Figure 10.**
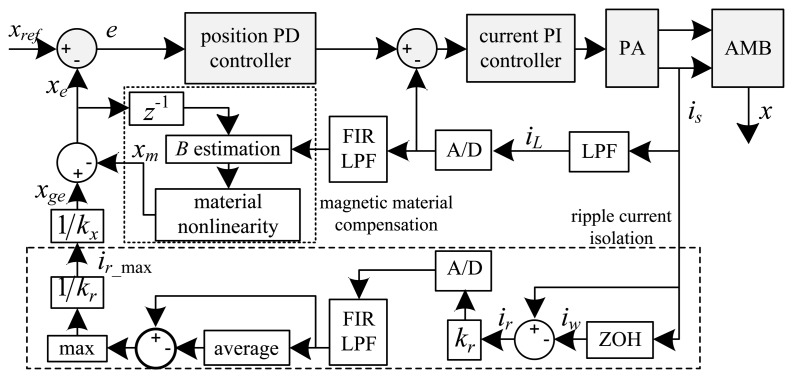
Configuration of the practical DCM position estimator.

In practice, PA switching noise degrades the signal-to-noise ratio, which makes direct application of Equation (5) difficult. Furthermore, since *i_r_* represents only a small percentage of the total current range, sampling resolution will be poor if *i_s_* is digitized directly. Therefore, a high-speed analog sample-and-hold (ZOH) circuit is implemented to isolate *i_r_* before digital sampling. Figure 10 presents the configuration of the total position estimation scheme.

The ZOH isolates the working point current (*i_w_*) at the beginning of the measurement cycle. Subtracting *i_w_* from the actual sensed current *i_s_* isolates *i_r_*. The ripple component is amplified by *k_r_* to the full range of the analog-to-digital (A/D) converter. Equation (5) is then implemented using the amplified *i_r_* in place of *i_s_*. The average of *k_r_i_r_* over the measurement cycle is subtracted from *k_r_i_r_* before taking the maximum and rescaling to obtain *i_r_*__max_. The estimated position is subtracted from the reference position to produce a position error, which is fed to the position controller. The current controller then generates the appropriate correction signal for the amplifiers using the control error. A low order finite impulse response (FIR) filter is implemented after the A/D converter to reduce the high frequency switching noise. The filter does introduce some unwanted phase-shift, but the cut-off frequency is chosen well beyond the sensor bandwidth at 20 kHz. An FIR filter is considered since a linear phase-shift for the frequency response is possible. Furthermore, classical position and current controllers are used to achieve stable suspension of the experimental AMB rotor, thereby demonstrating its feasibility for industrial application.

### 3.4. Modulation Self-Sensing Implementation

**Figure 11.**
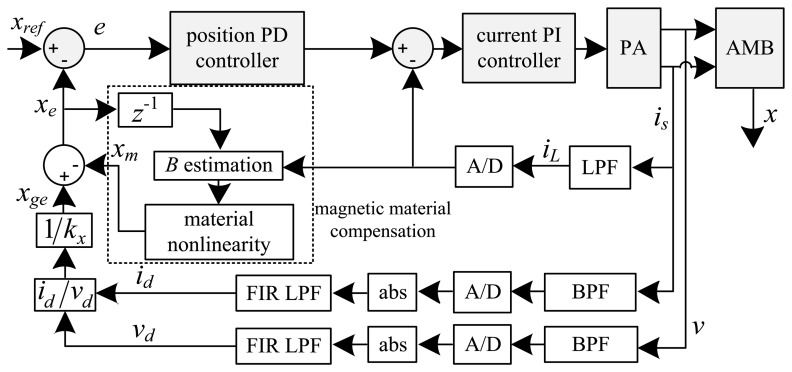
Configuration of the digital modulation position estimator.

Figure 11 shows a digital implementation of the modulation self-sensing approach used for comparison [3]. The signals are passed through analog BPFs before being digitized via a 1 MHz A/D converter. The BPFs isolate the fundamental components to improve sampling resolution. After digitization, envelope detection determines the ideal absolute value functions of the fundamental components and shifts the position information to low frequencies. LPFs then select only the low frequency baseband signals of interest. The digital filtering procedure is implemented in a digital signal processor. The analog BPF constitutes a second order switched capacitor filter with a pass-band. of 4 kHz and center frequency of 20 kHz. The 500th order FIR LPF has a cutoff frequency of 600 Hz, pass-band of 300 Hz, and a stop-band of 3,500 Hz.

## 4. Self-Sensing Performance Evaluation

### 4.1. Position Estimator Static Performance

**Figure 12.**
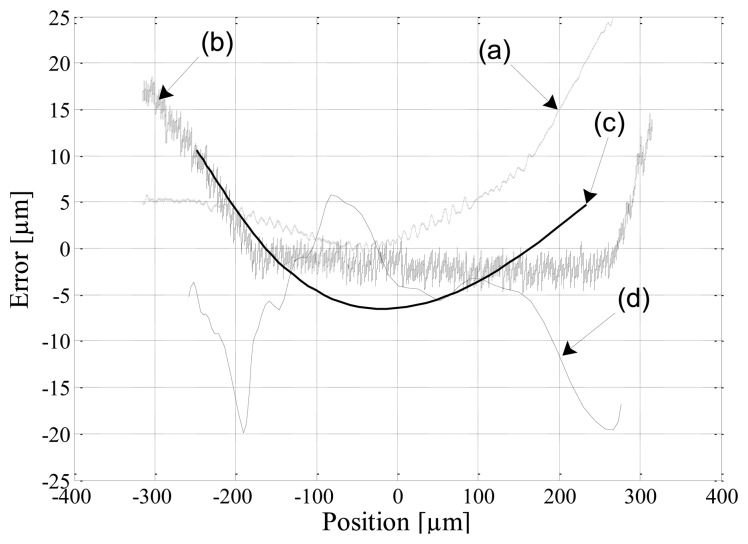
Simulated and experimental static position errors. Simulated: (a) modulation, (b) DCM; Experimental: (c) modulation, (d) DCM.

The static performance of the position estimators are judged in terms of sensor linearity for static position disturbances and currents. The desired position is linearly varied from −250 μm to 250 μm under open loop conditions with a constant bias current of 3 A. Figure 12 shows the errors between the simulated estimated positions compared to the output of the TSM, and a comparison between the experimental estimated positions and the reference sensors. In the figures, the following referencing notation is used: (a) simulated modulation self-sensing; (b) simulated DCM self-sensing; (c) experimental modulation self-sensing; and (d) experimental DCM self-sensing.

The results presented in Figure 12 compare favorably for DCM and modulation self-sensing. The experimental results for both cases show that the estimated positions differ less than 7 μm from the measured signal for rotor displacements in the range ±150 μm. The difference in the simulated and experimental trends in Figure 12 is mainly attributed to a mismatch between the simulation and the experimental implementation in terms of the magnetic nonlinearity compensation function. The effect of such a mismatch will be noticeable at the magnetic flux density extremes, which in this case coincide with the rotor displacement extremes. The proposed DCM self-sensing approach shows excellent linearity considering the nonlinear effects of the magnetic material as well as duty cycle variations.

### 4.2. Position Estimator Dynamic Performance

**Figure 13.**
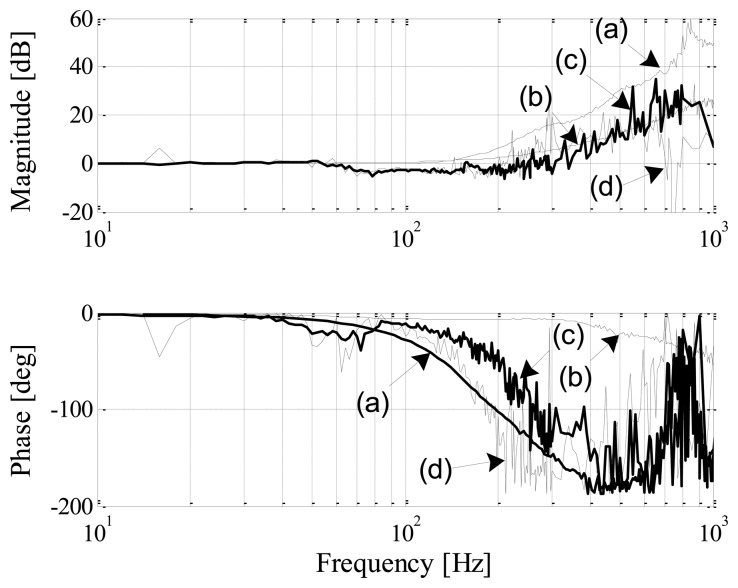
Frequency response of the simulated and experimental position estimators. Simulated: (a) modulation, (b) DCM; Experimental: (c) modulation, (d) DCM.

Figure 13 shows the frequency response of both the simulated and the experimental estimated positions with regard to the reference position. The AMB is perturbed with a 10 μm peak-to-peak sinusoidal position reference at different frequencies.

Ideally, the frequency response must have a magnitude of one and a phase of zero [3]. A reduced phase-shift is expected from the DCM method due to the fact that demodulation filters are largely excluded. In the modulation method the presence of the BPFs and FIR LPFs within the sensor bandwidth introduces large phase-shifts. Most noticeable from Figure 13 is the superior simulated phase response of the DCM method confirming the expected reduced phase-shift advantage. The practical phase, however, gradually increases for frequencies up to 400 Hz. The additional phase-shift observed in the practical results is mainly attributed to non-idealities in the analog ZOH circuit resulting in an amplitude disturbance during the negative slope of the current. For frequencies above 400 Hz, Figure 13 shows that the practical phase-shift of the DCM method is noticeably less if compared to the modulation approach. Additionally, the simulated and experimental results show an improvement in gain at high frequencies for DCM self-sensing.

### 4.3. Position Estimator Robustness

**Figure 14.**
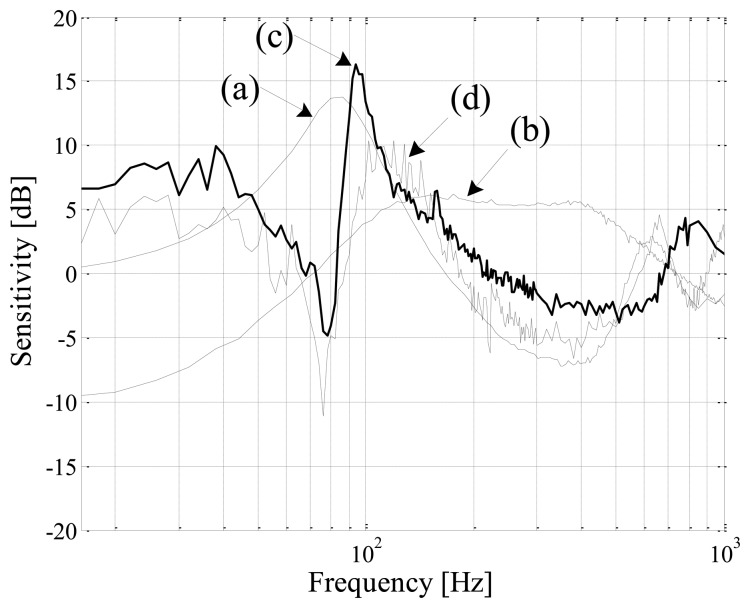
Simulated and experimental input sensitivity functions. Simulated: (a) modulation, (b) DCM; Experimental: (c) modulation, (d) DCM.

Magnetic bearings are inherently unstable and require feedback control to operate in a stable equilibrium [18]. The sensitivity function evaluates the robustness of the AMB control for parameter variations and disturbance forces. However, established robustness indicators do not yet exist for self-sensing AMBs [11,19]. The analysis is performed according to ISO 14839-3, which documents the sensitivity analysis for AMBs with standard displacement sensors. The rotor is suspended with the estimated position, after which a 7 μm peak-to-peak sinusoidal position reference with varying frequency is applied. The tests comprise stationary rotor conditions to facilitate evaluation of AMB performance independent from factors such as rotor circularity and unbalance that come into effect when the rotor is spinning. Figure 14 shows the sensitivity functions when the rotor is suspended with either the simulated or experimental estimated position.

The experimental curve yields a peak sensitivity of 10.3 dB for DCM self-sensing. According to the peak sensitivity zone limits [18], the experimental AMB is categorized in Zone B, rendering it possible for unrestricted long-term operation. Furthermore, a peak sensitivity of 10.9 dB is obtained in [3] for a low current (1.2 A) decoupled AMB employing the modulation approach. For the experimental digital modulation self-sensing implementation, a peak sensitivity of 16.3 dB is recorded. The results presented show a marked improvement in robustness for a high current magnetically coupled AMB that is attributable to DCM self-sensing.

Table 2 presents a summary of the frequency response results, showing the corner frequencies where deviations in gain and phase occur, as well as the peak sensitivity for each technique. From the table it is clear that the expected advantages of the DCM method could be realized in simulation, with minimal phase disturbance up to 1 kHz and a peak sensitivity of 6.2 dB. A comparison of the achieved experimental results with the theoretical limits as proposed by [7] therefore warrants further investigation.

### 4.4. Magnetic Cross-Coupling

Magnetic cross-coupling has the potential to significantly degrade self-sensing performance [15]. The influence of cross-coupling is practically quantified by clamping the rotor in the reference position, while applying the following actuation. The bottom vertical coil is supplied with a 3 A bias current while applying DCM self-sensing via alternate measurement cycles. The top coil is supplied with a 3 A bias current with a 5 A sinusoidal current at 160 Hz around the bias level for two cases; with a 50% (case 1) and varying (case 2) duty cycle during the measurement cycles. The estimated positions for the two cases are compared to actual position measurements.

**Figure 15.**
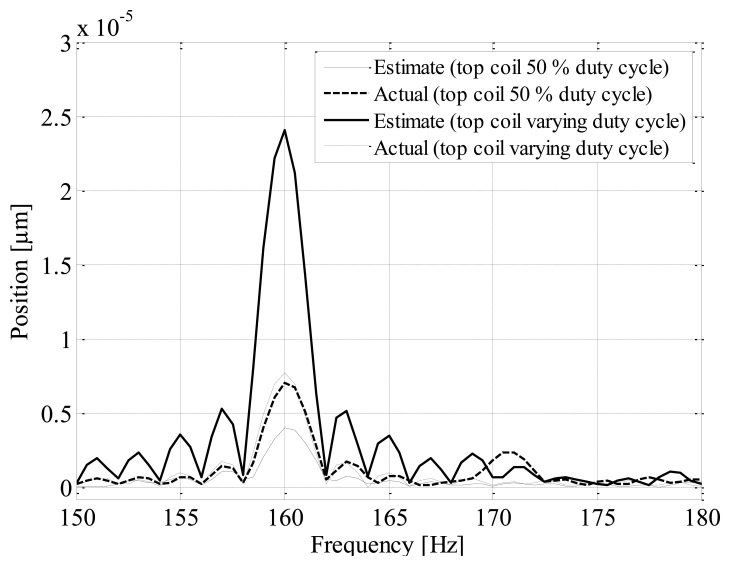
FFT position plots illustrating magnetic cross-coupling effects.

Figure 15 presents FFT plots of the estimated and actual positions for the cases described. The figure shows that for case 2 cross-coupling effects cause an error of approximately 300% when compared to the actual position. The results demonstrate that for the case where a 50% duty cycle is applied to the top coil during the measurement cycle, the effect of cross-coupling is partly attenuated. Similar results for different bias currents and frequencies confirm this observation [13].

## 5. Conclusions

This work presents the DCM approach for self-sensing AMBs. The proposed method is realized via a compact integrated PA that facilitates stable suspension of the experimental AMB rotor in one DOF. Position estimation is accomplished using only the measured current ripple of the sensing bearing coil. A novel switching method is proposed to reduce nonlinear modulation effects associated with voltage duty cycle change. The results indicate that phase-shift introduced by demodulation filters greatly influences self-sensing stability and bandwidth. The DCM approach employs minimal filtering in the demodulation path of the estimator, thereby minimizing additional phase-shift in the position estimates.

The DCM self-sensing AMB is evaluated in terms of static and dynamic performance. The linearity results show good agreement between the reference and estimated rotor displacement. In addition, the simulated and experimental gain of the DCM estimator compare favorably. However, some discrepancies are observed at high frequencies, which are mainly attributed to the unmodeled dynamics of the current ripple extraction circuit, as well as the high frequency switching noise in the experimental system. Although the improvements observed in the practical results are limited, the simulated results clearly highlight the performance advantages of the proposed method. Evaluation of the sensitivity function indicates that the robustness of AMB control using DCM self-sensing is satisfactory for unrestricted long-term operation. The proposed switching method minimizes the influence of magnetic cross-coupling on the position estimates without mechanical separation of the bearing coils, thereby reducing manufacturing costs.

The high current practical implementation of the DCM method for AMB control demonstrates feasibility for industrial application. However, self-sensing AMB dynamic performance is still limited compared to dedicated position sensors due to the duty cycle limitation imposed. Future directions of research will aim to improve the current ripple extraction methodology (eliminating the analog ZOH phase effect), as well as digital signal processing that enhance the signal-to-noise ratio of the practical estimator.

## Figures and Tables

**Table 1.** Experimental magnetic bearing and self-sensing parameters.

**Symbol**	**Quantity**	**Value**
*f_S_*	PWM switching frequency	20 kHz
*V_p_*	Switching voltage	50 V
*i_L_*	Maximum control current	10 A
*i*_0_	Bias current	3 A
*i_r__*_max_	Maximum current ripple	400 mA
*g*_0_	Nominal air gap length	0.676e−3 m
*N*	Coil turns	50
*R*	Coil resistance	0.2 Ω
*L*_0_	Nominal coil inductance	5.2 mH
*μ*_0_	Permeability of free space	4π × 10^−7^ H/m
*A*	Pole face area	0.616e−3 m^2^
*μ_r__*_max_	Relative magnetic permeability	4,000
*f*_LPF_	LPF cutoff frequency	5 kHz
*l_ax_*	Axial bearing length	44.358e−3 m
*r_r_*	Journal inner radius	15.875e−3 m
*r_j_*	Journal outer radius	34.95e−3 m
*r_p_*	Stator pole radius	35.626e−3 m
*r_c_*	Stator back-iron inner radius	60e−3 m
*r_s_*	Stator outer radius	75e−3 m
*w*	Pole width	13.89e−3 m
*K_P_*	Proportional constant (position controller)	10,000
*K_D_*	Derivative constant (position controller)	25
*K_P_*	Proportional constant (PA controller)	0.7
*K_I_*	Integral constant (PA controller)	0.01
*k_x_*	Conversion constant	156.25e−9 A/m

**Table 2.** Summary of self-sensing dynamic performance.

**Self-Sensing**	**0 dB (Hz)**	**20 dB (Hz)**	**0 Degree (Hz)**	**180° Phase Peak (Hz)**	**Sensitivity Peak (dB)**
Simulation					
(a) Modulation	150	400	50	400	13.7
(b) DCM	100	750	400	50° @ 1 kHz	6.2
Experimental					
(c) Modulation	300	550	100	450	16.3
(d) DCM	200	450	100	400	10.3
